# An exploratory study of challenges and successes in implementing adapted family-based treatment in a community setting

**DOI:** 10.1186/s40337-018-0228-9

**Published:** 2018-12-03

**Authors:** Ellen Astrachan-Fletcher, Erin C. Accurso, Setareh Rossman, Susan F. McClanahan, Gina Dimitropoulos, Daniel Le Grange

**Affiliations:** 1 Insight Behavioral Health Centers, Chicago, IL USA; 2 Department of Psychiatry and UCSF Weill Institute of Neurosciences, University of California, 401 Parnassus Avenue, Box F-0984, LPPI Room 368, San Francisco, CA 94143 USA; 3 Department of Psychology, Clark University, Worcester, MA USA; 4 Faculty of Social Work, University of Calgary, Calgary, AB Canada; 5 Hotchkiss Brain Institute Member of the Mathison Centre for Mental Health Research & Education, Calgary, Canada; 6 Emeritus Professor of Psychiatry and Behavioral Neuroscience, The University of Chicago, Chicago, IL USA

**Keywords:** Implementation, Family-based treatment, Dialectical behavior therapy, Anorexia nervosa, Adolescents

## Abstract

Although family-based treatment (FBT) is accepted as the first-line treatment for adolescent anorexia nervosa, studies show that it is infrequently used by clinicians in community settings. To elucidate some of the barriers to implementing this evidence-based treatment, mixed (quantitative and qualitative) methods were used in this exploratory study to examine therapist experiences with FBT. Twelve clinicians (*N* = 12) at a community treatment center retrospectively reported on their experiences with FBT training and supervision in FBT. A subset of clinicians (*n* = 7) additionally completed a structured interview about their experiences in using FBT. Results demonstrate that therapists endorsed certain common misconceptions about FBT prior to training, but that negative beliefs about FBT decreased after its implementation in their setting. These findings suggest that increased education about evidence-based treatments may diminish negative stereotypes about such treatments, which may ultimately increase their uptake in community settings. Sustainability of FBT is discussed in the context of how this community setting incorporated FBT principles into their ongoing clinical practice.

## Plain English summary

Involving families to support their teenage offspring with anorexia nervosa toward weight restoration is usually seen as the best treatment for this patient population. Yet, this treatment is seldom available in settings outside tertiary treatment programs. This study explored some of the barriers encountered by therapists when attempting to implement this family-based treatment in a community setting. Results show that while therapists endorsed certain beliefs that might interfere with using this family-based treatment in every day practice, these beliefs decreased after training and supervision in this treatment. Increased education about effective treatments may go some way toward making it easier for therapists to provide these treatments in settings outside of tertiary specialist centers. It is clear though that more research in this area is required.

## Introduction

Family-based treatment (FBT) is the leading empirically-supported outpatient treatment for medically stable adolescents with anorexia nervosa (AN) [16]. FBT engages parents and families as the primary resource to help patients reach weight restoration and return to their adolescent developmental trajectory. It follows three clearly defined phases (i.e., weight restoration under parental control, returning this control to the adolescent, and introducing adolescent developmental concerns in the absence of overwhelming eating disorder symptoms), within 18–20 treatment sessions [17, 18]. FBT produces favorable weight restoration and symptom reduction outcomes for adolescents with AN [13, 20]. However, remission (defined as > 94% of median Body Mass Index [mBMI] *plus* within one standard deviation of the Eating Disorder Examination [EDE] Global Score) at the end-of-treatment is only achieved in about 40% of adolescents [19]. While relatively little is known about who FBT works for or not, some moderators of outcome have indeed been identified, such as eating disorder related obsessionality and eating disorder specific psychopathology (e.g., [14, 21]).

Despite evidence that FBT is efficacious [19], anecdotally at least, it would appear that it is not widely implemented in the clinical world outside of tertiary medical centers [6]. This is consistent with research showing that several evidence-based interventions for childhood psychiatric disorders are rarely implemented in community-based clinical settings [24]. There is encouraging preliminary data that FBT can be successfully utilized in a private practice setting in order to achieve weight restoration [8]. In a recent study, Goldstein et al. [8], FBT was utilized in the care of 75 consecutive families attending a private practice, with 46% achieving full weight restoration at end of treatment, while 11% of the sample failed to achieve at least 85% mBMI. However, documented and/or systematic efforts to implement FBT in community-based settings, at least in the United States, remain sparse.

A variety of challenges may contribute to this research-practice gap ([3–6]; Kimber, [5]). For example, in one study from this group [3], a purposeful sample of 40 community-based clinicians who provide treatment to young persons with AN, the authors conducted in-depth interviews that described several obstacles to implementing FBT. Barriers to implementation identified by this group include intervention-specific factors (e.g., high demand on families and therapists, lack of involvement of dietician, required weekly weighing), organizational factors (e.g., administrative support, adequate space for family meals), interpersonal factors (e.g., previous training and therapy experience), patient and family factors (e.g., parental mental health and motivation), systemic factors (e.g., geographic location, lack of physician knowledge about eating disorders), and illness factors (e.g., co-occurring mental illness) [3]. This study, and other investigations by the same group, highlight many of the challenges we face in disseminating and implanting FBT in community-based settings. Further study is needed to better understand effective dissemination strategies as well as training approaches to further improve evidence based practice in community settings.

Given prior work underscoring several obstacles to the successful dissemination and implementation of FBT in community-based settings [6], the aim of the current study was to explore the challenges of implementing FBT according to clinicians at a large community-based setting. The study examined therapists’ *beliefs* and *experiences* while implementing an adaptive form of FBT in order to gain a better understanding of potential barriers to implementation.

## Method

### Study context

Our study was a collaboration between a clinical research program at an academic medical center and a national fee-for-service specialty treatment center, both located in a large US Midwestern city. At the time of the study, the treatment center offered intensive outpatient, partial hospitalization, and residential programs for eating disorders, mood and anxiety disorders. The present study was conducted at the main treatment center site, as well as at two associated satellite sites, and is part of a larger study regarding the implementation of a DBT “skills-enhanced” FBT in this setting [1]. All study procedures were approved by the institutional review boards at The University of Chicago and the University of California, San Francisco.

### Skills-enhanced FBT and therapist training

Prior to the study, clinic staff had utilized Dialectical Behavior Therapy [15] in their treatment of eating disorders. While there is limited evidence to suggest the suitability of DBT for an adolescent AN population, it is evident that several parents and teens do struggle with DBT related issues, such as distress tolerance and poor mood regulation. Acknowledging these clinical areas in this patient population, in conjunction with their existing DBT skills-set, integrating these aspects with FBT seemed a feasible avenue forward in this particular setting. Therefore, to improve the “fit” of FBT within this context, clinic staff collaborated with the researchers to modify standard FBT [18] given their DBT expertise. The resulting adaptation of FBT (called “skills-enhanced FBT”), included 19 sessions comprising a course of FBT (15–16 sessions) plus four skills-focused sessions based on those taught in DBT. The latter were incorporated within the first 6 weeks of delivering FBT. Core principles of FBT, as per the manual [18], include 1) appreciation for the strength and gravity of the eating disorder, which renders the adolescent incapable of exerting healthy control over her/his eating behavior, 2) a non-blaming approach toward both the parents *and* the adolescent, 3) a firm emphasis on early and rapid weight restoration to promote recovery, and 4) intensive parental involvement in supporting their adolescent through the process of weight restoration. FBT borrows from several domains in the broader family therapy literature, such as structural [22], strategic [10], systemic [23], and narrative family therapy [25]. FBT is divided into three phases: Phase 1 is almost exclusively concerned with weight restoration; Phase 2 is engaged with transitioning control of eating back to the adolescent in a developmentally appropriate fashion, and Phase 3 introduces adolescent developmental issues, in the absence of the eating disorder, and termination. DBT-based skills-focused sessions focused on four core skills 1) mindfulness, 2) validation, 3) distress and 4) emotion regulation [15].

Therapists were trained by ECA in a two-day workshop that addressed key FBT interventions through instruction, modeling, and role-play, and discussed how to integrate the skills within the FBT model. Therapists were also provided with the original (non-adapted) FBT manual [18]. A weekly one-hour group supervision meeting was provided by ECA and DLG for the duration of the active treatment component of the larger study.

## Methodology

A mixed-methods design [11] was chosen to report on both quantitative and qualitative study data. This mixed-methods approach was employed to expand on the quantitative findings by adding open-ended questions to qualitatively capture clinicians’ experiences of implementing skills-enhanced FBT in the community. The qualitative findings generated from the open-ended questions were used to contextualize and deepen the understanding of the quantitative findings about therapists’ beliefs about FBT.

### Participants

Therapists were invited to participate in data collection relevant to this manuscript if they had received FBT training from authors DLG or ECA. Twelve therapists were considered eligible because they had either attended a FBT training workshop led by DLG or participated in at least six FBT supervision sessions led by DLG and ECA. All therapists had participated in weekly group supervision calls (50% 20+ times, 42% 11–20 times, and 8% 6–10 times), and over half had also attended the FBT training workshop. The rest of the therapists had attended FBT training workshops before their collaboration on this study. Seven of the twelve therapists had provided FBT within the context of the larger study referred to above [1]. On average, participants had utilized FBT with about half dozen cases (*M* = 6.83, *SD* = 5.20);

### Procedure

The data collected for this study focused on therapist perceptions of FBT, which were gathered after completion of the active treatment component of the study. All therapists (*N* = 12) anonymously completed a two-part questionnaire created for this study. In part one of this questionnaire, therapists were presented with ten possible beliefs about FBT, and asked (1) retrospectively to report on how much they agreed with each belief before learning FBT, as well as (2) on how much they agreed with the belief now (See Table 1 for the FBT beliefs/ideas). These ten beliefs, considered to be common misconceptions about FBT, were chosen by the authors based on informal feedback from study participants and the greater clinical community. Results were recorded on a 5-point Likert scale from *not at all*, *slightly, somewhat, very much*, and to *totally*. In part two of this questionnaire, therapists were asked to assess to what extent certain activities (e.g., “experience treating families using FBT”) changed their overall perception of FBT. Change in perception was reported on a 7-point Likert scale ranging from − 3 (*very much worsened perception*), no change, or + 3 (*very much improved perception*).

**Table 1:** Therapists Perception of FBT at time of Training^a^ and at time of Delivery

	Training^b^ M (SD)	Delivery^b^ M (SD)	Sign p	Cohen’s d
FBT is a heartless treatment	1.25 (0.62)	1.08 (0.29)	.44	0.248
FBT is traumatic for the adolescent	2.17 (1.12)	2.00 (0.85)	.59	.0.119
Adolescents recover from anorexia if treated with FBT	3.17 (0.58)	3.33 (0.65)	.50	−0.191
FBT can only works with well-functioning families	2.92 (1.38)	2.50 (1.00)	.32	0.245
FBT is traumatic for parents	2.08 (0.90)	2.17 (0.94)	.82	−0.067
FBT does not care about the adolescent’s feelings	1.83 (1.19)	1.25 (0.62)	.17	0.434
FBT is force feeding the adolescent	2.17 (1.12)	1.67 (0.99)	.19	0.338
FBT discourages therapists to use their therapeutic skills	2.50 (1.24)	1.50 (0.67)	.004	0.707
FBT does not value therapeutic relationships	1.33 (0.89)	1.17 (0.39)	.59	0.165
FBT is the most evidence-based treatment available	3.00 (1.21)	4.08 (0.67)	.012	−0.783

*FBT* Family-based treatment, *M* Mean, *SD* Standard Deviation; ^a^Collecting data about the time of Training was done retrospectively; ^b^Likert Scale from *Not at All* (1) to *Totally* (5)

Additionally, therapists who had been involved in the larger study ([1]; *N* = 7) anonymously responded to 11 open-ended written questions regarding their experience implementing skills-enhanced FBT. These prompts asked about therapists’ experiences implementing FBT with DBT skills, including their perceptions of helpful resources and common challenges. Examples of these prompts include; *What was the experience like learning a totally new treatment*?, *At what point did you begin to feel more comfortable using this new treatment?, At what point did it feel more natural*?, *What helped you in acclimating to the new treatment?, What did you find most helpful about supervision*?, and *What were some of the biggest obstacles this experience*?

### Data analysis

Mixed (quantitative and qualitative) methods were used in this exploratory study to examine therapist’s experiences about implementing enhanced FBT. Descriptive statistics and paired t-tests with SPSS V24 were used to examine beliefs about FBT and changes in those beliefs over time. Common codes from the subset of seven therapists were extracted from open-ended questions about their experiences implementing FBT to complement the quantitative findings.

Using Summative Content Analysis [9], qualitative responses were read in their entirety and then re-read by two research associates with the aim of becoming immersed in the responses to the open-ended questions. Summative content analysis was used to identify and compare the frequency and the usage of written words described by the therapist participants in this study. We used pre-defined codes such as beliefs about delivering FBT and skilled enhanced FBT in the community and implementation issues to understand the usage of key words provided by participants. We counted how commonly therapist participants used the same codes (or key words to describe a similar experience. The research team was especially interested in the changes in perceptions over time. Each associate independently created a code-book of phrases or sentiments emerging from the responses. The associates met to discuss and merge code-books. The final code-book was used to independently code the responses and to identify consistency and discrepancy in the usage and occurrence of key words to describe the use of FBT and enhanced FBT from the data. They further cross-checked the codes with quotes from the data to ensure accuracy of summative content findings which added rigor to our study.

## Results

### Common beliefs about FBT

Participating clinicians (*N* = 12) endorsed several beliefs about FBT related to appropriateness, acceptability, and fidelity that might be present among the eating disorders treatment community.

### Appropriateness of FBT

Belief in the appropriateness of the treatment, or its “fit” with the treatment population was assessed through three different beliefs/ideas. Asking therapists to look back at the time of their FBT training, they now (at the time of the current study inquiry) endorsed *somewhat* believing that “Adolescents recover from AN when they are treated with FBT,” which did not significantly change after training and supervision. The absence of a significant change may be related to the fact that the study site had typically offered only higher levels of care; therapists had treated many adolescents who had reportedly ‘tried’ FBT before, unsuccessfully, which may have influenced therapists’ beliefs about the effectiveness of FBT. However, we do not know if these adolescents were actually treated by clinicians knowledgeable about FBT. Furthermore, most therapists reported believing *somewhat* before training that “FBT is the most empirically supported treatment for adolescent AN.” After training and supervision, they reported a significant increase in their agreement with this statement (*t* = − 3.03, *p* = .012, Cohen’s d = − 0.783), showing greater appreciation for the evidence supporting FBT.

On the other hand, a majority of the therapists reported believing *slightly* to *somewhat* that “FBT can only be done with well-functioning, intact families,” and this was a deterrent to their desire to use FBT with the varied and complex population presenting to their clinic. There was no statistically significant change in this belief after training and supervision. While clinicians reported learning that FBT need not *only* be done with well-functioning families, they also reported that family dysfunction sometimes posed a challenge to administering skills-enhanced FBT. Therapists (*n* = 3) discussed difficulty “staying the course” when families did not “buy-in” to treatment, or therapists themselves had doubts about whether FBT was appropriate. For example, when families demonstrated clear marital discord, individual parental emotional struggles, poor general family functioning, or adolescent problems outside of the eating disorder, some clinicians struggled to consistently redirect the focus to the primary priority of refeeding the adolescent. During training, therapists learned that FBT acknowledges other issues but indicates that these issues should be addressed only after the adolescent’s life is no longer in danger. However, marital issues and single parent families were noted in weekly supervision meetings as potential feasibility concerns, particularly when one parent was primarily responsible for supporting the family financially. That said, two therapists commented on their difficulties in two-parent families when the father neither attended sessions nor actively participated in re-nourishment efforts at home.

### Acceptability and Fidelity of FBT

Acceptability of FBT, or perception by therapists that FBT is agreeable, was evaluated by examining therapists’ retrospective endorsement of seven beliefs at the time of their FBT training, and current endorsement of these same beliefs upon completing providing FBT. Overall, study clinicians did not endorse the belief that “FBT does not care about the feelings of the adolescent” or that “FBT is a heartless treatment.” However, clinicians did report *slightly* believing that “FBT is traumatic for the adolescent” and that “FBT is traumatic for the parents,” none of which changed significantly after training and supervision. One therapist provided poignant insight: *“There is a difference between my beliefs about FBT in theory and in practice. In theory I do think that FBT is supposed to care about the adolescent’s feelings and be able to be done with families that are less than optimally prepared; however, in practice I felt these things were less easily attainable. And while I don’t believe it is inherently a traumatic treatment, I do think it has the potential to feel very traumatic at times, especially to the adolescent.”*

Prior to training and supervision, clinicians *slightly* endorsed the belief that “FBT encourages force feeding adolescents,” with no significant decrease in this belief after training and supervision. Throughout training and supervision clinicians were made aware that, while FBT emphasizes the focus on eating at this time, physical force is never permitted. Parents “taking control” of the eating disorder means they communicate consistently that nothing is more important than eating. For instance, parents are encouraged to limit their child’s participation in recreational activities involving high energy expenditure, such as sports or dance, until the child is well nourished or approaches a healthy weight.

Fidelity issues did arise for clinicians as they seemed to struggle with the perceived lack of flexibility that is inherent to the FBT model. Specifically, we examined the belief that “FBT limits therapists’ ability to use their therapeutic skills.” While the clinicians on average did endorse this belief *somewhat* before training, they endorsed the belief significantly less after training and supervision (*t* = 3.63, *p* = .004, Cohen’s *d* = 0.707). Study clinicians often described subscribing to the “art of psychotherapy,” and they initially described not feeling able to use their clinical instincts and intuition while adhering to FBT. It was clear that clinicians struggled with the tension that occurs between strict fidelity to FBT and the desire to meet the family where they are at, perhaps not appreciating the flexibility that is akin to remaining adherent to the FBT model, but still meeting a family clinically where they are at. Over time, however, instincts and intuition began to coincide with FBT protocol as clinicians had more experience seeing how much weight restoration helped to decrease the adolescent’s suffering. Therapists (*n* = 6) generally reported feeling comfortable working within the FBT model after treating two to four families. However, this sense of comfort was also nuanced. For instance, while one therapist endorsed FBT as “*fantastic for weight restoration*”, s/he goes on to add that*, “it doesn’t really address skills for coping once the adolescent is weight restored and the ED cognitions still remain*.” Another study clinician also alluded to the same somewhat mixed picture of being comfortable with FBT, on the one hand saying: “*I believe assessing the appropriateness of FBT with certain families/parents is a crucial part of the process that has not received enough attention*.” Yet, she goes on to say: “*I have found some families/parents are simply unable or unwilling to carry out what is needed for FBT to be an effective process, and it can sometimes be difficult to determine when that may be the case. Supervision did help address this*.”

### Perceived barriers and facilitators to implementing skills-enhanced FBT

In addition to the therapist beliefs explored above, participating clinicians qualitatively reported on various advantages and challenges to implementing skills-enhanced FBT based on their experiences in the larger study (c.f., [1]). Therapists reported on the degree to which each of six factors (i.e., training, supervision [external versus internal], experience treating families, and discussions with FBT providers [internal versus external]) related to training, supervision, and experience administering treatment affected their perceptions of FBT. On a Likert scale of − 3 to + 3, therapists reported that all six factors improved their perceptions of FBT, and none worsened their perceptions of FBT (all *M*’s 1.43 to 2.50). This suggests that therapists overall believed that experience with FBT led to more positive beliefs about FBT. Therapists endorsed external supervision as the factor that most improved their perceptions of FBT (*M* = 2.50). In open-ended responses, seven of eight therapists highlighted supervision as one of the most useful tools supporting implementation; six also referenced the FBT manual as a helpful resource, and all noted the importance of practice.

Although learning and implementing skills-enhanced FBT entailed various challenges, at the end of the study therapists also reported finding both the learning experience and the treatment modality to be valuable. One therapist reported “…*FBT training and its use … was very helpful and beneficial to the families involved*.” Another commented, “… *despite the feelings of fear that came with learning FBT, I felt extremely fascinated by such a radical way of dealing with a very severe illness. The distinctness of the approach helped me feel invigorated by the work and hopeful that my patients would reach recovery, and I think that hopefulness is a vital piece in FBT sessions if it is effectively balanced with graveness*.” Therapists also noted a desire to incorporate FBT principles into other aspects of their treatment practice. For example, one therapist reflected, “*I have used the skills I learned in my FBT trainings to treat a variety of eating disorder presentations, because the concepts coming out of the FBT framework and the comprehensive depiction of the way the illness pervades the person have fostered a very rich understanding of the disease and how it needs to be addressed*.”

### Additional challenges to implementing skills-enhanced FBT

In addition to the ten common beliefs about FBT that were explored via the therapist questionnaire, 7 therapists anonymously responded to 11 open-ended questions regarding their experience implementing skills-enhanced FBT. Several therapists (*n* = 5) described the process of learning a new treatment as *overwhelming*, *nerve-wracking*, *stressful*, *anxiety-provoking*, and associated with *feelings of fear*, although the process was also described as *intriguing*, *exciting*, *stimulating*, and *energizing*. A quarter of therapists noted initially struggling with *not feeling like an expert* and *needing to learn* to become “*the authority for the family on eating disorders*.” Another challenge arose regarding collaborating with additional treatment providers, many of whom lack knowledge about the treatment modality (i.e., FBT). One therapist explained that “*it was sometimes challenging to implement consistently due to other treatment providers being involved [in the patient’s care] who were not as familiar with FBT*.”

Therapists also noted some philosophical differences with the FBT model, such as the role of dieticians in treatment. In standard FBT, as outlined in the clinician manual [18], registered dietitians are not included as principal members of the treatment team because parents are viewed as the experts in refeeding their child. In contrast, at facilities traditionally offering higher levels of care, registered dietitians are instrumental consultants to parents and providers who serve in an educational role. At the end of the current study, one therapist described that she struggled with “*the perception that a dietician has no role in FBT ... it felt that way before FBT group supervision and still feels that way now*.” Although therapists did have opportunities to discuss the role of dieticians during the weekly supervision calls, their reports of continued frustration or confusion about the role of dieticians suggest that differences between this particular philosophy espoused by FBT and that held at the treatment center might have posed some challenges to treatment acceptability.

Fidelity to the treatment model, assessed informally during weekly supervision, also suffered at times. Interestingly, fidelity of the DBT component of the study appeared to suffer more than fidelity of the standard FBT protocol (c.f., [1]). It appeared that as clinicians became more comfortable and familiar with FBT, some therapists noticed that their older, more comfortable treatment modalities were at times forgotten. As they focused on adhering to FBT, at times they reported forgetting that one purpose of the larger study was to help families use DBT skills that they learned while receiving FBT (c.f., [1]). Whereas this focus helped clinicians develop FBT skills, they expressed distress when realizing that they neglected to use what previously came more naturally, i.e., their DBT skills.

## Discussion

FBT has been shown to be the most efficacious treatment for adolescent AN [13, 16], but anecdotally it is not widely used in community-based clinical settings, especially in the United States [6, 12]. The current exploratory study is one of only a few to investigate potential barriers and enablers to the implementation of FBT outside academic settings. We retrospectively assessed clinician beliefs about FBT at the time of FBT training and after the implementation of FBT. We examined clinician beliefs related to the appropriateness and acceptability, feasibility, and sustainability of FBT that might impact implementation. We also explored clinicians’ experiences related to challenges while implementing skills-enhanced FBT. Finally, we illustrated how FBT has been integrated into in a community treatment center’s ongoing care, demonstrating one example of FBT’s sustainability.

Clinicians endorsed several beliefs and experiences that contributed to discomfort or lack of confidence in using FBT. Their belief that some adolescents recover from AN when treated with FBT or that only some aspects of recovery are aided by FBT is not inconsistent with the literature, which demonstrates that only one quarter to one half of adolescents achieve full remission at the end of treatment [19], and that there are only small improvements in weight and shape concerns at 12-month follow-up [2]. In addition, clinicians expressed concerns about its appropriateness in complex families, which is not surprising given that family dynamics and communication patterns significantly impact the therapeutic work. Clinicians raised concerns about families with more limited resources to supervise re-nourishment, who had parents who were less able to support each other’s efforts, who themselves expressed concerns about the appropriateness of this treatment approach, or who were unwilling to engage in this treatment. To date, research has not elucidated a broad range of family characteristics that could inform adaptations or identified clear predictors of potential treatment failure, which may be important to overcoming this barrier to treatment, for both clinicians and families.

Clinicians also endorsed believing that FBT could be “traumatic” for the adolescent and/or parents, which did decrease with supervision. This perception that FBT “feels traumatic” is understandable given the complexities of treating a fatal, yet typically ego-syntonic, disease. Ultimately, FBT takes the stance that weight restoration process is primary in order to ensure the safety of the adolescent, and charges caregivers with the task of ensuring consistent weight gain. Clinicians reported high confidence that FBT achieved this aim better than other treatments but believed that the method through which weight restoration occurs could be traumatic for both the adolescent and parents(s). Indeed, FBT can lead to an elevated emotional response in the familial context, since parents are encouraged to prioritize the adolescent’s eating, even if this means deprioritizing other important life activities such as school.

The results of our study showed that supervision, education, training, and increasing experience using FBT might have contributed to growing comfort, acceptability, as well the sustainability of this intervention. Therapists (all of whom had experience in DBT) were encouraged to see patients’ suffering in light of the DBT principle that “the only way out of hell is through suffering.” It was also understood that the eating disorder is the source of distress, rather than the treatment, and that returning to normal eating is the first step to interrupting the emotional control the eating disorder has over the adolescent. Nevertheless, these beliefs persisted at some level and suggest that FBT’s strict focus on re-nourishment is viewed as an important limitation of FBT. Clinicians expressed difficulty with the families’ distress around the re-nourishment process and commented on the absence of techniques to specifically address the cognitive aspects of recovery. Additional concerns arose around FBT being an “overwhelming” and “anxiety-provoking” treatment to learn and implement, and worries that clinicians were not expert enough to deliver this treatment, suggesting that clinicians new to FBT would benefit from intensive consultation, particularly early on in implementing FBT. Feasibility was also challenged by other providers who were not knowledgeable about FBT (e.g., collaborating with pediatricians who did not support treatment) or providers who were “left out” of treatment (e.g., dieticians). These data suggest that implementation would be compromised unless FBT more directly addresses these limitations, or training/supervision is more effective in changing these beliefs that may inadvertently impact implementation (e.g., clinicians decelerating weight gain in order to balance compassion for the adolescent and/or parents).

Another dilemma in the implementation of FBT is that this intervention fosters a primary therapeutic relationship with the parents/family rather than the young person with AN. Indeed, in the beginning of FBT treatment, adolescents may resent their therapist for coaching their parents to set limits with the eating disorder and focus the family primarily on helping the adolescent to restore weight. Because of this, we expected that clinicians might be skeptical about providing a treatment that they did not think maintained the therapeutic alliance with the patient as a priority. However, on average therapists did not endorse a belief that “FBT does not value therapeutic relationships.” Instead, clinicians seemed to view the attitude towards adolescents as ‘tough love’ (i.e., ‘tough’ towards the eating disorder and ‘love’ towards the adolescent). Reminding clinicians in supervision to conceptually separate the eating disorder from the adolescent probably helped them appreciate the role of this approach. Of note, and contrary to a belief held by many clinicians, prior research findings suggest that patients do report relatively strong therapeutic alliances in FBT [7].

### Sustainability and ongoing use of FBT

The treatment center chose to integrate FBT principles into their ongoing therapy offerings, after completion of the larger study. To do this, the research team created a program and handbook for FBT at higher levels of care for adolescents with AN and their families who are regarded not a good fit for outpatient FBT. When doing outpatient FBT, strong expressions of parental burnout appeared to frequently precede referrals to higher levels of care. For example, single parents with more than one child and/or with little extra support often tried to balance the needs of their child with AN with other needs (e.g., paying bills). For such families, a potential benefit of FBT in higher levels of care is to enable parents to get a break from what they report as unrelenting arguments with the eating disorder, while remaining in control of the weight restoration process.

Once in a higher level of care, families often turned over re-nourishment to the “professionals” and parents lost a sense of empowerment. This seemed to have had a significant impact on sustainability of the treatment and the progress families made in treatment. As parental empowerment is a necessary component of FBT, the treatment center’s response to this dilemma was to base the adolescent Partial Hospitalization (7 days a week, 8 h a day) and Intensive Outpatient Programs (3–5 days a week, 3 h a day) on the fundamental assumptions in FBT.

To integrate FBT into its higher levels of care, the treatment center created “family days” that emphasized FBT principle that “*neither parents nor the adolescent are to blame for the illness”*. Rather, empowering parents is instrumental in the process of adolescent recovery. Therefore, families are required to participate in treatment at all levels of care and they are responsible for providing food or selecting provided foods for their child, even during program hours, as well as participating in meals at least once per day. Family days also provides education in externalizing the eating disorder, and the need to join the family against the eating disorder. Finally, psychoeducation is provided around the fact that re-nourishment must be well-underway before one can expect the adolescent participate more effectively in therapy targeting eating disorder cognitions. As an example of ‘well underway,’ an adolescent who started FBT around 80% mBMI, and has progressed through Phase 1 and most of Phase 2, and now is quite well > 90% mBMI, would typically be able to more effectively participate in her/his own treatment as the clinician moves toward Phase 3. This scenario will of course be contingent on the age/developmental stage of a particular adolescent and the extent to which the ED has interrupted regular adolescent development.

Other facets of FBT-informed higher levels of care are the inclusion of a registered dietician who empowers parents by providing nutritional education and consultation (but not a meal plan), a two-week intensive review period in order to evaluate this treatment track’s effectiveness for each new patient, adolescent engagement in individual therapy, DBT skills groups to help them find their wise minded values to guide recovery, and family-based groups focused on distress tolerance skills to aid in the process of re-nourishment.

### Limitations

Several limitations to our exploratory study should be considered. The sample size was modest, and the questionnaire was developed by the research team, and administered to therapists retrospectively due to the research question being developed part-way through the larger study (c.f. [1]). Given the potential for recall bias in this case, it is therefore possible that therapists may have misreported their initial endorsement of certain beliefs about FBT, perhaps deeming them to be artificially similar to their current beliefs. That said, this study was exploratory and modest in nature, and findings should be interpreted in this context.

## Conclusion

This study suggests that several of the perceived barriers to implementing FBT can be successfully negotiated by confronting widely held FBT beliefs with FBT realities. Novel ways to increase access to FBT training should be explored, as our study provides only preliminary support for the idea that access to training in FBT might make it more likely that clinicians will want to use this treatment despite earlier misgivings. Also, given the finding that therapists struggled with the exclusion of dieticians from treatments, a more systematic examination exploring whether and how dieticians can support FBT is warranted. Finally, it might be helpful to incorporate FBT into higher levels of care to reach those families who are reluctant or exhausted to engage in traditional outpatient treatment. In this way, families are prepared to step-down to a lower level of care and perhaps better prepared to engage in FBT with an understanding of its core principles. Future research to address these questions should be more systematic and utilize a larger sample.

## References

[1] Accurso EC, Astrachan-Fletcher E, O’Brien S, McClanahan SF, Le Grange D (2018). Adaptation and implementation of FBT enhanced with dialectical behavior therapy skills for anorexia nervosa in community-based specialist clinics. Eat Disord.

[2] Accurso EC, Ciao AC, Fitzsimmons-Craft EE, Lock JD, Le Grange D (2014). Is weight gain really a catalyst for broader recovery? The impact of weight gain on psychological symptoms in the treatment of adolescent anorexia nervosa. Behav Res Ther.

[3] Couturier J, Kimber M, Jack S, Niccols A, Van Blyderveen S, McVey G (2013). Understanding the uptake of FBT for adolescents with anorexia nervosa: therapist perspectives. Int J Eat Disord.

[4] Couterier J, Kimber M, Sztamari P (2013). Efficacy of family-based treatment for adolescents with eating disorders: a systematic review and meta-analysis. Int J Eat Disord.

[5] Couterier J, Kimber M, Jackson S, Niccols A, Van Blyderveen S, McVey G (2014). Using a knowledge transfer framework to identify factors facilitating implementation of family-based treatment. (2014). Int J Eat Disord.

[6] Couterier J, Lock J, Kimber M, McVey G, Barwick M, Niccols A, et al. Themes arising in clinical consultation for therapists implementing family-based treatment for adolescents with anorexia nervosa: a qualitative study. J Eat Disord. 2017;5:28. 10.1186/s40337-017-0161-3 PMC5582386 28878927

[7] Forsberg S, LoTempio E, Bryson S, Fitzpatrick KK, Le Grange D, Lock J (2013). Therapeutic alliance in two treatments for adolescent anorexia nervosa. Int J Eat Disord.

[8] Goldstein M, Murray SB, Griffiths S, Rayner K, Powkowka J, Batemen JE, et al. The effectiveness of family-based treatment for full and partial adolescent anorexia nervosa in an independent private practice setting: clinical outcomes. Int J Eat Disord. 2016;49(11):1023–2016. 10.1002/eat.22568 27270494

[9] Hsieh HF, Shannon SE (2005). Three approaches to qualitative content analysis. Qual Health Res.

[10] Haley J, Hoffman L (1973). Techniques of family therapy.

[11] Johnson RB, Onwuegbuzie AJ, Turner LA (2007). Toward a definition of mixed methods research. J Mixed Methods Res.

[12] Kimber M, Couturier J, Georgiades K, Wahoush O, Jack SM (2014). Decision-making processes for the uptake and implementation of family-based therapy by eating disorder treatment teams: a qualitative study. Int J Eat Disord.

[13] Le Grange D, Hughes E, Court A, Yeo M, Crosby R, Sawyer S (2016). Randomized clinical trial of parent-focused treatment and family-based treatment for adolescent anorexia nervosa. J Am Acad Child Adolesc Psychiatry.

[14] Le Grange D, Lock J, Agras WS, Moye A, Bryson SW, Jo B, Kraemer HC (2012). Moderators and mediators of remission in family-based treatment and adolescent focused therapy for adolescent anorexia nervosa. Behav Res Ther.

[15] Linehan M (1993). Skills training manual for treating borderline personality disorder.

[16] Lock J (2015). An update on evidence-based psychosocial treatments for eating disorders in children and adolescents. J Clin Child Adolesc Psychol.

[17] Lock J, Le Grange D (2013). Treatment manual for anorexia nervosa: a family-based approach.

[18] Lock J, Le Grange D (2013). Predicting recovery in eating disorders. Int J Eat Disord.

[19] Lock J, Le Grange D. Family-based treatment: Where are we and where should we be going to improve recovery in child and adolescent eating disorders? Int J Eat Disord. 2018. In press. 10.1002/eat.22980 30520532

[20] Lock J, Le Grange D, Agras WS, Moye A, Bryson SW, Jo B (2010). Randomized clinical trial comparing family-based treatment to adolescent focused individual therapy for adolescents with anorexia nervosa. Arch Gen Psychiatry.

[21] Lock J, Agras WS, Bryson S, Kraemer H (2005). A comparison of short- and long-term family therapy for adolescent anorexia nervosa. J Am Acad Child Adolesc Psychiatry.

[22] Minuchin S, Baker L, Rosman BL, Liebman R, Milman L, Todd TC (1975). A conceptual model of psychosomatic illness in children. Family organization and family therapy. Arch Gen Psychiatr.

[23] Palazzoli MS, Boscolo L, Cecchin GF, Prata G (1974). The treatment of children through brief therapy of their parents. Fam Process.

[24] Weisz JR, Ng MY, Bearman SK (2014). Odd couple? Re-envisioning the relation between science and practice in the dissemination-implementation era. Clin Psychol Sci.

[25] White M, Epston D (1990). Narrative means to therapeutic ends.

